# Hats off to 20S proteasome substrate discovery

**DOI:** 10.1038/s44320-024-00028-7

**Published:** 2024-03-12

**Authors:** Taylor R Church, Anna Brennan, Seth S Margolis

**Affiliations:** 1 Department of Biological Chemistry, The Johns Hopkins University School of Medicine, Baltimore, MD 21205 USA; 2 Solomon H. Snyder Department of Neuroscience, The Johns Hopkins University School of Medicine, Baltimore, MD 21205 USA

**Keywords:** Post-translational Modifications & Proteolysis, Proteomics

## Abstract

The substrates of uncapped free 20S proteasomes have remained underexplored. In their recent study, Sharon, Picotti and colleagues (Pepelnjak et al, 2024) develop a proteomics-based method (PiP-MS) and comprehensively explore 20S proteasome substrates in the human proteome.

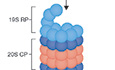

## Teaser

Uncapped free 20S proteasomes are abundant and active in cells, and yet their degraded intracellular protein substrates have remained underexplored. In their recent study, Sharon and colleagues (Pepelnjak et al, 2024) developed an advanced proteomics method (PiP-MS) to comprehensively explore 20S proteasome substrates in the human proteome.

**Figure 1:**
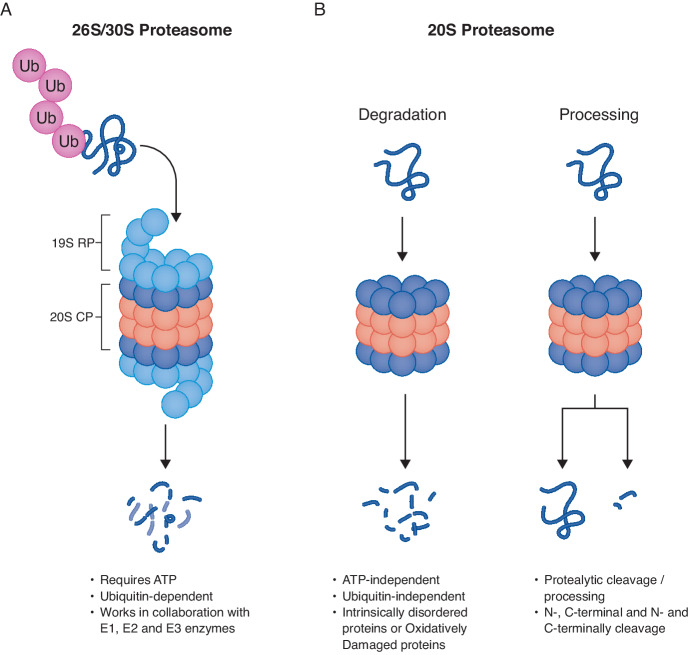
Overview of mechanisms that distinguish capped and uncapped proteasomal degradation. (**A**) Schematic of capped (26S/30S) proteasome degradation. The 20S core particle (CP) can associate with one (26S) or two (30S) regulatory caps (RP), illustrated in light blue. Capped proteasome degrades substrates in an ATP and Ubiquitin-dependent manner in collaboration with E1, E2, and E3 ligases. (**B**) Degradation: The uncapped (20S) proteasome degrades substrates in an ATP- and Ubiquitin-independent manner. Generally, substrates of the uncapped proteasome are intrinsically disordered proteins and/or oxidatively damaged proteins, although these substrates remain poorly understood. Pepelnjak et al, (2024) develop a proteasomal induced proteolysis-mass spectrometry (PiP-MS) method to systematically distinguish substrates of the uncapped proteasome. Processing: using PiP-MS, Peplnjak et al, identify 20S CP substrates that undergo specific C- and N-terminal cleavage.

Protein translation is influenced by many factors, including environmental stressors and the age of the cell. Continuous translation leads to a pool of proteins that eventually exhibit a diverse history of molecular experiences such as post-translational modifications (e.g. phosphorylation or ubiquitylation) and even structural changes that render the molecule inactive. Moreover, translation may generate molecules that are not folded properly or that are not needed by the cell for reasons that remain unknown. As the accumulation of misfolded or damaged protein molecules can have detrimental consequences, cells have evolved mechanisms to remove them via the proteasome, a large macromolecular complex that mediates protein degradation (Coux et al, 1996). The core proteasome, referred to as the 20S, is made up of 28 subunits that form a barrel-like shape with a central pore accessible to select substrates for degradation. However, the proteasome is considerably more complex than it appears. Around 50% of our 20S proteasome complexes can associate with additional protein complexes to form a modified 20S. For example, the 19S cap, which is made up of 19 additional proteins, can associate on one or both sides of the 20S and form a 26S or 30S, respectively (Ben-Nissan and Sharon, 2014; Ciechanover, 1998; Ciechanover and Schwartz, 1998). These capped proteasomes are specialized so that they can degrade intracellular proteins that have been uniquely tagged with ubiquitin by any of the 600 E3 ligases in our cells. These tagged proteins make their way to the proteasome whereby the 19S recognizes this ubiquitin-tagged protein, binds to it, deubiquitinates it, begins to unfold it using associated ATPases, and feeds it into the barrel of the 20S for its degradation (Fig. 1). Besides the 19S, other 20S-associated cap complexes can mediate ubiquitin-independent opening of the proteasome and targeting of proteins to the 20S for degradation (Fig. 1). Much of our understanding of 20S-mediated degradation in mammalian cells has been achieved by using proteasome inhibitors that either non-covalently or covalently interfere with the catalytic sites of the 20S proteasome (Coux et al, 1996; Goldberg, 2012). While highly effective for analyzing protein degradation mechanisms, proteasome inhibitors have not been able to effectively distinguish between the uncapped 20S and capped 20S proteasomes. In fact, roughly 50% of the proteasomes in our cells remain without a cap (Fabre et al, 2013; Türker et al, 2023). There is an ongoing perspective that the capped proteasome is the only complex that is actively degrading intracellular protein substrates and that the uncapped 20S is predominantly latent. However, the catalytic subunits of the 20S core are always active, and a major limiting factor in substrate degradation is access of a substrate to the core. By that measure, uncapped 20S is likely degrading intracellular proteins, but it remains unknown to what extent this is essential to biology relative to the capped proteasome. Besides approaches to suppress ubiquitination, there are currently no tools that allow for direct inhibition of the capped versus uncapped 20S. One approach to address this important question is to look at what is being degraded by these two complexes. We now know that interfering with global ubiquitination does not eliminate 20S proteasome-dependent degradation and cells can continue to survive and function. However, the targets of this ubiquitin-independent proteasome degradation have remained largely undiscovered. Moreover, it is not clear whether proteasome-dependent cellular and physiological effects are caused solely by altered levels of the proteasomal client proteins (through proteasome-mediated degradation), or whether these effects are caused, at least in part, by the altered levels of the cleavage products of the client proteins. Indeed, proteasomes produce peptide fragments that are 6 to 18 amino acids long, and while 97% of these peptides are broken down into single amino acids, roughly 3% of these peptides are utilized within seconds of production in cellular signaling and other unknown pathways (Coux et al, 1996; Kisselev et al, 1999). More recently, we discovered an uncapped 20S that operates by degrading intracellular proteins in the neuron directly into extracellular signaling peptides (Türker et al, 2024). Taken together, discovering substrates and developing tools to identify 20S-derived peptides will enhance our understanding of many important and emerging biological phenomena and will establish the foundation for understanding 20S-specific degradation pathways.

In their recent study, Sharon and colleagues (Pepelnjak et al, 2024) developed new approaches to both identify the 20S substrate proteins and to better understand how oxidized 20S proteasomes interfere with its degradation capacity. To start, they developed PiP-MS (proteasome-induced proteolysis coupled to mass spectrometry). This approach uses a combination of cell lysates and purified 20S proteasomes followed by proteomics to identify the proteins that are able to be degraded by the free 20S. Through rigorous quantitative analysis, they revealed a comprehensive list of proteins that are substrates of the 20S proteasome. They showed that even upon suppression of all ubiquitination, these proteins are still subject to proteasomal degradation, consistent with the 20S working to target these proteins for turnover in cells. These results validate their approach and substantiate a first list of potential 20S substrates. Subsequent to these efforts, using PiP-MS, they showed that oxidative stress does indeed reduce the activities of 20S-dependent protein degradation. This result is important for the field as it has remained unclear to what extent 20S proteasome substrates may be subject to stress-induced changes in proteasome function. An aggregate evaluation of 20S substrates illustrated that many of the targets were disordered DNA- and RNA-binding proteins. Whether this reveals the cellular location of the 20S-induced degradation will likely have to be determined on a substrate-to-substrate basis. It is possible that many of the substrates could reside in the cytosol or nucleus, two locations where 20S proteasomes reside. Another very intriguing aspect of their work is that they identify several proteins that are not completely degraded but are rather proteolytically processed (Fig. 1). This opens an important area of investigation and establishes the intriguing question of how this proteolytic processing may contribute to the proteins’ function and to what extent the proteasome may work to regulate these events. Overall, Sharon and colleagues have provided the scientific community with new information regarding many 20S substrates, resulting peptides, and new techniques that can be further applied to a variety of cell systems and questions. Their work sets a foundation for future 20S proteasome pathway biology and discovery.
